# Current-Aware Temporal Fusion with Input-Adaptive Heterogeneous Mixture-of-Experts for Video Deblurring

**DOI:** 10.3390/s26010321

**Published:** 2026-01-04

**Authors:** Yanwen Zhang, Zejing Zhao, Akio Namiki

**Affiliations:** Department of Mechanical Engineering, Chiba University, Chiba 263-8522, Japan; 24wd4101@student.gs.chiba-u.jp (Z.Z.); namiki@faculty.chiba-u.jp (A.N.)

**Keywords:** video deblurring, current–aware temporal fusion, heterogeneous experts, training strategy

## Abstract

In image sensing, measurements such as an object’s position or contour are typically obtained by analyzing digitized images. This method is widely used due to its simplicity. However, relative motion or inaccurate focus can cause motion and defocus blur, reducing measurement accuracy. Thus, video deblurring is essential. However, existing deep learning-based video deblurring methods struggle to balance high-quality deblurring, fast inference, and wide applicability. First, we propose a Current-Aware Temporal Fusion (CATF) framework, which focuses on the current frame in terms of both network architecture and modules. This reduces interference from unrelated features of neighboring frames and fully exploits current frame information, improving deblurring quality. Second, we introduce a Mixture-of-Experts module based on NAFBlocks (MoNAF), which adaptively selects expert structures according to the input features, reducing inference time. Third, we design a training strategy to support both sequential and temporally parallel inference. In sequential deblurring, we conduct experiments on the DVD, GoPro, and BSD datasets. Qualitative results show that our method effectively preserves image structures and fine details. Quantitative results further demonstrate that our method achieves clear advantages in terms of PSNR and SSIM. In particular, under the exposure setting of 3 ms–24 ms on the BSD dataset, our method achieves 33.09 dB PSNR and 0.9453 SSIM, indicating its effectiveness even in severely blurred scenarios. Meanwhile, our method achieves a good balance between deblurring quality and runtime efficiency. Moreover, the framework exhibits minimal error accumulation and performs effectively in temporal parallel computation. These results demonstrate that effective video deblurring serves as an important supporting technology for accurate image sensing.

## 1. Introduction

Image sensing refers to measuring physical quantities by analyzing images captured by vision sensors. These quantities include the position, shape, motion, and other scene attributes. Because image sensing offers convenient acquisition, rich information, and non-contact measurement, it has been widely used in many applications. Chang et al. [1] presented a safety warning system for motorcycles, Katsamenis et al. [2] conducted work on real-time patrol vehicle monitoring, and Yao et al. [3] developed an instance segmentation method for galloping data of high-speed railway overhead contact system conductors. These studies are all based on image sensing, and their target objects are often in high-speed motion. However, the relative motion between the camera and the target or inaccurate focus can cause motion blur and defocus blur, reducing the measurement accuracy. Based on this observation, video deblurring is considered an effective way to mitigate this problem. In this context, a practical video deblurring algorithm should offer effective deblurring, fast computation, and broad applicability. In recent years, with the rapid advancement of deep learning, video deblurring algorithms [4] have achieved remarkable progress.

According to the type of input data, mainstream deep learning deblurring methods can be divided into two categories: single-frame methods that do not rely on additional or neighboring frames [5,6,7,8,9,10,11] and methods that leverage additional or neighboring frames, including reference-based methods [12,13,14,15], recurrent methods [16,17,18,19], sliding window-based methods [20,21,22], and parallel methods [23,24]. The choice of input type directly affects the degree of feature reuse, the richness of the observational information, the feasibility of parallel computation, the memory consumption, the extent of error accumulation, and the adaptability to complex scenarios.

Feature reuse can effectively reduce redundant computation [25]. With sufficient input features such as sharp pixels from neighboring frames, the network can effectively exploit spatial and temporal information, which enhances its ability to handle complex deblurring scenarios. Recurrent and parallel methods support feature reuse and provide sufficient input features. However, parallel methods consume large memory because they deblur multiple frames simultaneously. In recurrent methods, either hidden states or previously reconstructed frames are used. Hidden states do not support temporal parallel computation, whereas previously reconstructed frames can be substituted, enabling parallel processing. To enable feature reuse, provide rich observational information, and allow efficient processing, the input of the deblurring framework should comprise the previously reconstructed frame, the current frame, and future frames. Therefore, during inference, we need to address two main challenges: (1) mitigating the error accumulation that arises from temporal sequential dependencies and (2) maintaining high performance while allowing parallel computation.

Single-frame video deblurring can be regarded as a special case of image restoration [6,26]. Both tasks emphasize feature extraction and reconstruction from a single frame. Single-frame methods are simple and efficient and do not suffer from error accumulation, but they lack information from additional frames and neighboring frames, therefore, perform poorly in complex blur scenarios. In contrast, the clear features or structures from additional frames, as well as the temporal dynamics and inter-frame dependencies from neighboring frames, can assist in deblurring the current frame, enabling effective handling of complex blur scenarios. Nevertheless, some single-frame methods [6,27] outperform some methods that utilize neighboring frames or additional frames on the GoPro dataset [6]. This result may be attributed to two distinct points: (1) From a structural perspective, based on Huang et al.’s research on screen content video quality enhancement [28], single-stream methods may suffer from interference caused by irrelevant features from neighboring frames, which hinders the network’s effective learning of the target frame. (2) From an algorithmic perspective, we observe that existing methods lack distinct processing for different frames, indicating that the features of the current frame and neighboring frames are not fully utilized. Note that these two points are distinct: The former emphasizes the issue of information interference caused by the single-stream structure, whereas the latter emphasizes that the lack of distinct processing leads to insufficient utilization of features.

Video frames exhibit varying levels of blur. Lightly blurred frames retain more details, which shallow modules can capture, while heavily blurred frames benefit from deeper modules to recover lost information. Adapting the network depth to input characteristics can improve the reconstruction quality and reduce the inference time. We argue that the Mixture-of-Experts (MoE) module [29] selects the most suitable expert from a set of experts with different depths based on the input features, enabling adaptive processing.

**Figure 1:**
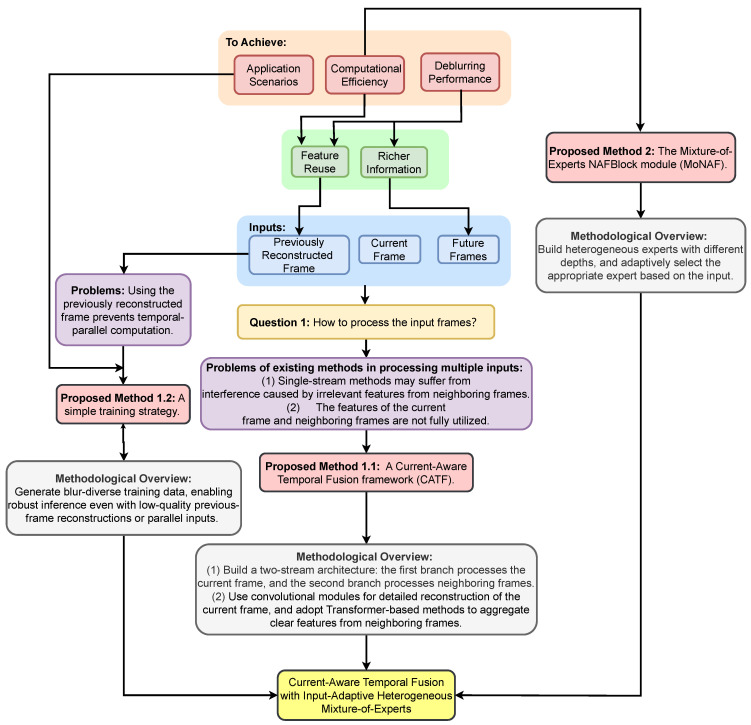
Motivation and design of our method.

Figure 1 provides an overview of the motivation and design rationale of our method. Based on this overview, our contributions are summarized as follows:

- To address the issues of insufficient utilization of the current frame and the interference from irrelevant features of neighboring frames in single-stream structures, this paper proposes a current-aware temporal fusion framework. A simple training strategy is used to achieve reasonable performance in both sequential and parallel computation.
- Considering the varying blur in video frames, this paper proposes a Mixture-of-Experts structure with shallow, medium, and deep modules, achieving high deblurring quality while reducing the inference time.
- Qualitative and quantitative experiments demonstrate that our algorithm performs well in both deblurring quality and inference speed in sequential computation. Furthermore, experiments confirm that the proposed framework exhibits minimal error accumulation during sequential inference and achieves satisfactory results in temporal parallel computation.

## 2. Related Work

### 2.1. Video Deblurring Methods

**Figure 2:**
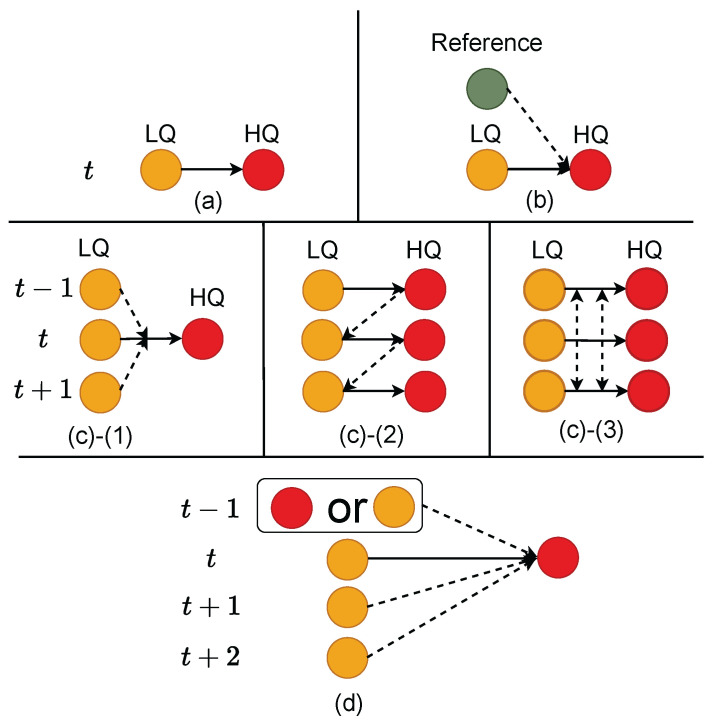
Illustrative comparison of different methods. (**a**) Single-frame-based method, (**b**) reference-based method, (**c-(1)**) sliding window-based method, (**c-(2)**) recurrent method, (**c-(3)**) parallel method, and (**d**) the proposed method. Yellow, red, and green circles denote low-quality input frames, high-quality output frames, and reference frames, respectively. t−1, *t*, t+1, and t+2 indicate frame indices. Dashed lines illustrate information fusion across frames. A more detailed explanation is provided in Section 2.1.

Single-frame-based methods [5,6,7,8,9,10] (Figure 2a) take the current frame as input to reconstruct a sharp image at that time step without using any additional information. Inspired by the physical degradation process, Li et al. [7] jointly learned image reblurring and deblurring in an end-to-end manner, capturing spatially adaptive degradation representations. Chen et al. [6] focused on network simplicity and efficiency by extracting the essential components of state-of-the-art methods and replaced or removed nonlinear activation functions. Single-frame-based methods are simple and efficient, with no cumulative error, but they do not use additional information and are limited in handling complex blur scenarios.

Reference-based methods [12,13,14,15] (Figure 2b) use clear features or structures from reference frames to assist in deblurring the current frame. Liu et al. [12] used the neighboring frames of ground-truth images as reference to assist in restoring blurry images. The results showed that different reference images could significantly affect the clarity of the output. Zou et al. [13] used images captured from different angles, times, or positions as references. The results showed that reference image blur affected the deblurring quality, and the degree of impact depended on the network structure. Li et al. [14] used a ranking correlation module to selectively extract useful features from reference images, improving robustness even when exemplars differed from the input. However, ablation studies showed that removing the correlation module or exemplar had little impact, indicating they did not contribute significantly. Reference-based methods can provide additional information to improve performance, and their effectiveness depends on the quality of the reference frame, which we consider entirely reasonable. However, high-quality reference frames should be easily obtainable without requiring a complex selection process.

**Figure 3:**
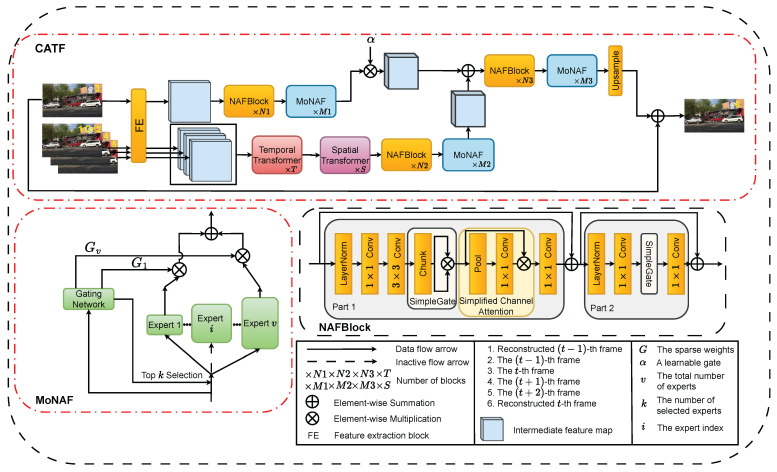
The architecture of the Current-Aware Temporal Fusion (CATF) framework. The CATF and MoNAF (red boxes) are proposed in this paper. CATF corresponds to Equations (1)–(12), and MoNAF corresponds to Equations (13) and (14). The CATF architecture first reconstructs the current frame using a convolutional module composed of NAFBlock and MoNAF, while reconstructing a single neighboring frame with a combination of Temporal Transformer, Spatial Transformer, NAFBlock, and MoNAF. In the Mixture-of-Experts NAFBlock module (MoNAF), the expert structures correspond to the NAFBlock: the deep expert is the complete NAFBlock, the medium expert corresponds to Part 1, and the shallow expert corresponds to Part 2.

Sliding window-based (Figure 2c-(1)), recurrent (Figure 2c-(2)), and parallel (Figure 2c-(3)) methods recover clear frames by fusing information from the current frame and its neighboring frames. Sliding window-based methods [20,21,22,30,31] take multiple consecutive frames as input and perform deblurring. Recurrent methods [16,17,18,32] sequentially propagate latent features or deblurred results from one frame to the next. Parallel methods [23,24] restore all frames in parallel. Due to differences in input and output, these three methods each have their own advantages and disadvantages, as shown in Table 1. Considering the diverse application scenarios of video deblurring, the challenge is to achieve ease of use, feature reuse, rich observational information, minimal error accumulation, support for parallel computation, and high processing speed.

As shown in Figure 2d, to achieve feature reuse, richer information, and improved computational efficiency, we extend sliding window-based methods to include the previously reconstructed frame, the current frame, and future frames. To enable temporal parallel computation, the previously reconstructed frame can be replaced by the previous frame. The remaining challenge is how to mitigate the error accumulation introduced by the previously reconstructed frame while still achieving satisfactory results during parallel computation.

### 2.2. Temporal Fusion

Sliding window-based methods focus on utilizing information from neighboring frames. Zhang et al. [34] utilized temporal–spatial attention module and frame channel attention module to capture temporal and spatial information from neighboring frames. Zhang et al. [20] utilized a spatio-temporal deformable attention module to capture sharp pixel information from consecutive video frames. Li et al. [35] built a correlation volume pyramid by matching pixels between the reference and neighboring frames. Cao et al. [21] proposed Temporal Transformers to aggregate clear features from neighboring frames and Spatial Transformers to reconstruct high-quality latent frames. Most sliding window-based methods [21,35,36] usually adopt a single-stream structure, which, according to [28], increases interference from irrelevant features in neighboring frames.

Recurrent methods focus on how to utilize and propagate hidden states or the previously reconstructed frame between frames. Zhong et al. [17] propagated hidden information through residual dense blocks and employed a global spatio-temporal attention to fuse temporal features. Ji et al. [18] used a memory bank to store blurry–sharp feature pairs, providing effective information for the propagation and fusion of hidden features in the bidirectional recurrent network. Lin et al. [32] used ConvLSTM [37] to update hidden features while propagating and updating the cell state across time, capturing long-range temporal feature variations. The hidden features were fused with the current frame features via simple element-wise addition. Zhu et al. [16] proposed a simple yet effective multi-scale bi-directional propagation module, designed to capture inter-frame information from unaligned neighboring hidden states across multiple scales, without requiring explicit alignment. Some methods [38,39] used the previously reconstructed frame as the input between frames. The feature processing in these methods is very similar to that of sliding window-based methods. Although recurrent methods inherently cannot perform temporal parallel computation, if the network is designed to allow the previously reconstructed frame to be replaced with an alternative input, the output may be affected but remains usable. In this case, data from different time steps can be processed in parallel, enabling temporal parallel computation.

Parallel methods [23,24] perform long-range feature interactions between frames to improve deblurring effectiveness, resulting in high memory usage.

We observe that some single-frame methods outperform certain methods that exploit neighboring frames. This may be because the latter methods [35,40] do not differentiate the roles of different input frames and instead extract features using the same approach, preventing full utilization of both the current and neighboring frame features.

## 3. Proposed Methods

As shown in Figure 3, this paper proposes a Current-Aware Temporal Fusion (CATF) framework, comprising a current-frame reconstruction branch and a neighboring-frame reconstruction branch. The collaborative design of the two branches alleviates interference from irrelevant features in neighboring frames, which is common in single-stream architectures. The features of the current frame are rich and can be directly used for detail reconstruction via convolutional modules, while the features of neighboring frames are first aggregated through Transformer-based modules to extract clear features before detail reconstruction. By employing differentiated feature extraction and a simple fusion strategy, the features of the current frame are fully utilized, with neighboring frame features providing corresponding assistance. Subsequently, we introduce a Mixture-of-Experts module [29] based on NAFBlocks (MoNAF) to adaptively select its structure according to the input, reducing the inference time without compromising the reconstruction quality. Finally, to alleviate the error accumulation arising from temporal sequential dependencies while supporting parallel computation, we adopt a training strategy that preserves the reconstruction quality of temporal sequences and ensures that parallel computation achieves comparably satisfactory results.

### 3.1. The Current-Aware Temporal Fusion Framework

Transformer and convolution are currently the two most popular network architectures. Multi-Head Self-Attention (MSA) facilitates the integration of global contextual information, whereas convolution excels at modeling local structures and fine details [41]. The CATF framework leverages the complementary strengths of both: for current-frame reconstruction, convolution modules are used to restore local features, while for the reconstruction of a neighboring frame, the Transformer module first integrates the clear features from neighboring frames, followed by convolution modules for local refinement. The input to the CATF framework consists of the previously reconstructed frame $R_{t-1}\in R^{3\times H\times W}$, the current frame $F_{t}^{\mathrm{input}}\in R^{3\times H\times W}$, and the subsequent two frames $F_{t+1}^{\mathrm{input}}\in R^{3\times H\times W}$ and $F_{t+2}^{\mathrm{input}}\in R^{3\times H\times W}$. The output is the reconstructed current frame $R_{t}$.

First, as shown in Figure 3, $R_{t-1}$, $F_{t}^{input}$, $F_{t+1}^{input}$, and $F_{t+2}^{input}$ are fed into the FE block for feature extraction. The FE block consists of three convolutional layers followed by LeakyReLU activations, producing the feature representations $R_{t-1}^{FE},F_{t}^{FE},F_{t+1}^{FE},F_{t+2}^{FE}\in R^{C\times \frac{H}{p}\times \frac{W}{p}}$.

Then, the current frame is independently processed using convolutional modules to extract high-frequency spatial features, where the convolutional modules are implemented as NAFBlocks [6]. The reconstructed feature of the current frame, denoted as $F_{t}^{C,\;\mathrm{out}}\in R^{C\times \frac{H}{p}\times \frac{W}{p}}$ is obtained as follows.

(1) $$\begin{array}{c} F_{t}^{C}=\mathrm{NAFBlock}{(F}_{t}^{FE}), \end{array}$$

(2) $$\begin{array}{c} F_{t}^{C,out}=\mathrm{MoNAF}\left(F_{t}^{C}\right), \end{array}$$

where NAFBlock(·) represents the NAFBlock, and MoNAF(·) is described in Section 3.2.

Next, the reconstruction of a neighboring frame employs a Temporal Transformer to extract clear temporal information from neighboring frames, while a Spatial Transformer captures spatial information for high-quality reconstruction. Subsequently, NAFBlock and the proposed MoNAF are applied to enhance the high-frequency components of the reconstructed frame. The reconstructed feature of a neighboring frame, denoted as $F_{t+1}^{N,out}\in R^{C\times \frac{H}{p}\times \frac{W}{p}}$ is obtained as follows.

(3) $$\begin{array}{c} {F_{t+1}^{TT}=G}_{TT}\left(Cat\left(R_{t-1}^{FE},F_{t+1}^{FE},F_{t+2}^{FE}\right)\right), \end{array}$$

(4) $$\begin{array}{c} F_{t+1}^{LT}\;=G_{ST}\left(F_{t+1}^{TT}\right), \end{array}$$

(5) $$\begin{array}{c} F_{t+1}^{N,out}=\mathrm{MoNAF}\left(\mathrm{NAFBlock}\left(F_{t+1}^{LT}\right)\right), \end{array}$$

where $G_{TT}$ and $G_{ST}$ denote the Temporal Transformer and the Spatial Transformer [21], respectively.

The main difference between the Temporal and Spatial Transformers lies in how they compute the Query, Key, and Value. In the Temporal Transformer, these are computed as $Q^{TT}\in R^{\left(w\times w\right)\times \left(\frac{H_{p}}{w}\times \frac{W_{p}}{w}\right)\times C}$, and $K^{TT},V^{TT}\in R^{\left(w\times w\times 3\right)\times \left(\frac{H_{p}}{w}\times \frac{W_{p}}{w}\right)\times C}$.

(6) $$\begin{array}{c} P^{TT}=\mathrm{Flatten}\left(Cat\left(R_{t-1}^{FE},F_{t+1}^{FE},F_{t+2}^{FE}\right)+{LPE}^{TT}\right), \end{array}$$

(7) $$\begin{array}{c} Q^{TT}=P_{t+1}^{TT}W_{q}^{TT},\;K^{TT}=P^{TT}W_{k}^{TT},\;V^{TT}=P^{TT}W_{v}^{TT}, \end{array}$$

where $H_{p}$ and $W_{p}$ represent $\frac{H}{p}$ and $\frac{W}{p}$, respectively. ${LPE}^{TT}\in R^{(w\times w\times 3)\times 1\times C}$ provides a 2D learnable positional encoding to enhance spatial positional information, and Flatten(·) converts patches into one-dimensional vectors. $P_{t+1}^{TT}\in R^{\left(w\times w\right)\times \left(\frac{H_{p}}{w}\times \frac{W_{p}}{w}\right)\times C}$ denotes the patch from the (t+1)-th frame used as the Query, indicating that only the features of the (t+1)-th frame are reconstructed. $W_{q}^{TT},W_{k}^{TT},W_{v}^{TT}\in R^{C\times C}$ are learnable linear projection matrices.

In the Spatial Transformer, the Query, Key, and Value are computed as $Q^{ST},K^{ST},V^{ST}\in R^{\left(w\times w\right)\times \left(\frac{H_{p}}{w}\times \frac{W_{p}}{w}\right)\times C}$.

(8) $$\begin{array}{c} P^{ST}=\mathrm{Flatten}\left(F_{t+1}^{TT}+{LPE}^{ST}\right), \end{array}$$

(9) $$\begin{array}{c} Q^{ST}=P^{ST}W_{q}^{ST},K^{ST}=P^{ST}W_{k}^{ST},V^{ST}=P^{ST}W_{v}^{ST}, \end{array}$$

where ${LPE}^{ST}\in R^{(w\times w)\times 1\times C}$ is the 2D learnable positional encoding, $P^{ST}\in R^{\left(w\times w\right)\times \left(\frac{H_{p}}{w}\times \frac{W_{p}}{w}\right)\times C}$ denotes the input patches, and $W_{q}^{ST},W_{k}^{ST},W_{v}^{ST}\in R^{C\times C}$ are the learnable linear projection matrices.

After that, $F_{t+1}^{N,out}$ is incorporated into $F_{t}^{C,out}$ through a simple and efficient fusion strategy, thereby mitigating artifacts introduced by alignment errors.

(10) $$\begin{array}{c} F_{t}^{M}\;=\;F_{t+1}^{N,out}+\alpha *F_{t}^{C,out}, \end{array}$$

where $\alpha \in R^{C\times 1\times 1}$ is a learnable weight.

Finally, we further reconstruct $F_{t}^{R}\in R^{C\times \frac{H}{p}\times \frac{W}{p}}$ from the fused features $F_{t}^{M}\in R^{C\times \frac{H}{p}\times \frac{W}{p}}$.

(11) $$\begin{array}{c} F_{t}^{R}=\mathrm{MoNAF}\left(\mathrm{NAFBlock}\left(F_{t}^{M}\right)\right), \end{array}$$

(12) $$\begin{array}{c} R_{t}=\mathrm{Upsample}\left(F_{t}^{R}\right)+\;F_{t}^{input}, \end{array}$$

where Upsample(·) progressively enlarges the feature maps through convolution and PixelShuffle operations.

### 3.2. The Mixture-of-Experts Module Based on NAFBlocks

Given that the degree of blur may vary across frames in a video, we propose a Mixture-of-Experts module [42] based on NAFBlocks (MoNAF). MoNAF extends the standard NAFBlock by introducing a heterogeneous Mixture-of-Experts mechanism. Each expert is constructed with a different depth, allowing the network to adaptively choose the most suitable one for each input, thereby ensuring both effective and efficient deblurring. As shown in Figure 3, the NAFBlock is divided into Part 1 and Part 2, where Part 2, Part 1, and the full NAFBlock act as shallow, medium, and deep experts, respectively. The function MoNAF(·) applied to $F_{t}^{C}$ is defined as follows.

(13) $$\begin{array}{c} \ell_{\text{Top-k}},\;i_{\text{Top-k}}=\mathrm{TopK}\left(\mathrm{Gate}\left(F_{t}^{C}\right),k\right), \end{array}$$

(14) $$\begin{array}{c} g_{t,r}=\mathrm{Softmax}{\left(\ell_{\text{Top-k}}\right)}_{r},\;r=1,\ldots ,k, \end{array}$$

(15) $$\begin{array}{c} G_{t,i}=\left\{\begin{array}{cc} g_{t,r}, & \mathrm{if}\;i=i_{\text{Top-k}}^{\left(r\right)}\;\mathrm{for}\;\mathrm{some}\;r,\\ 0, & \mathrm{otherwise}, \end{array}\right.\;i=1,\ldots ,v, \end{array}$$

(16) $$\begin{array}{c} F_{t}^{C,\mathrm{out}}=\sum_{i=1}^{v}G_{t,i}\;E_{i}\left(F_{t}^{C}\right), \end{array}$$

where Gate(·) maps $F_{t}^{C}$ to expert logits over *v* experts. TopK(·,k) selects the top-*k* experts and returns their logits $\ell_{\text{Top-k}}\in R^{1\times k}$ and their indices $i_{\text{Top-k}}=\left(i_{\text{Top-k}}^{\left(1\right)},\ldots ,i_{\text{Top-k}}^{\left(k\right)}\right)\in {\left\{1,\ldots ,v\right\}}^{k}$, ordered in the same way as $\ell_{\text{Top-k}}$. The vector $g_{t}=\left(g_{t,1},\ldots ,g_{t,k}\right)\in R^{1\times k}$ contains the normalized gating weights over these *k* experts, so that $\sum_{r=1}^{k}g_{t,r}=1$. $G_{t}\in R^{1\times v}$ denotes the final sparse gating weights, and $F_{t}^{C,\mathrm{out}}$ is obtained as the weighted sum of expert outputs. $E_{i}\left(\cdot \right)$ denotes the *i*-th expert.

### 3.3. The Training Strategy

We generate a new training dataset by linearly combining the input blurred frames with the ground truth at different ratios. This new dataset includes frames with varying levels of blur, which are used as the reconstructed previous frame inputs during training. Since the network learns to handle highly blurred frames during training, it can achieve good results during inference even when the previous frame’s reconstruction is of low quality or when the previous frame is used instead of the reconstructed result in parallel computation. The generated dataset is computed as follows.

(17) $${\tilde{R}}_{s,t}=A_{t}F_{s,t}^{input}+\left(1-A_{t}\right)\times GT_{s,t}$$

where *s* denotes the *s*-th video segment, with s∈{1,2,…,S}, and *t* denotes the *t*-th frame within a video segment, with t∈{1,2,…,T}. $A_{t}$ represents the weight, where a smaller $A_{t}$ corresponds to a lower level of image blur. Within the same video segment, the values of $A_{t}\in \left\{0.1,0.2,\ldots ,0.9\right\}$ are uniformly assigned to all frames in a random order.

The training is divided into three stages. In the first stage, covering the first $E_{1}$ epochs, a dataset generated by linear combinations is used as the previous-frame reconstruction input, enabling CATF to handle previous-frame reconstructions with varying degrees of blur. In the second stage, the pre-trained network obtained after $E_{1}$ epochs generates ground-truth reconstructions, which are used as the new previous-frame reconstruction dataset to enhance the network’s adaptation to sequential temporal computation. In the third stage, the previous-frame reconstruction dataset is updated again at epoch $E_{2}$. Through these three stages, CATF achieves excellent deblurring performance under sequential temporal computation while maintaining good results under parallel temporal computation.

### 3.4. Loss Function

#### 3.4.1. Reconstruction Loss

The reconstruction loss guides the network to learn the input–output correspondence in pixel space. The calculation is as follows.

(18) $$\begin{array}{c} L_{\mathrm{rec}}=\sqrt{{∥R-GT∥}^{2}+\epsilon }, \end{array}$$

where *R* denotes the reconstructed image, GT denotes the corresponding ground truth, and ϵ=0.001 is used to stabilize training.

#### 3.4.2. Perceptual Loss

The perceptual loss uses the Mean-Squared Error (MSE) in the VGG [43] feature space to encourage structures, textures, and edges that better match human perception. The calculation is as follows.

(19) $$\begin{array}{c} L_{Per}=\mathrm{MSE}\left({\mathrm{VGG}}_{h}\left(R\right)-{\mathrm{VGG}}_{h}\left(GT\right)\right) \end{array}$$

Here, ${\mathrm{VGG}}_{h}$ represents the feature map produced by the *h*-th layer of the VGG network.

#### 3.4.3. MoE Loss

The MoE loss [42] is used to balance the utilization of each expert. The calculation is as follows.

(20) $$\begin{array}{c} {\mathrm{GTL}}_{bi}=\left\{\begin{array}{cc} 1, & G_{bi}>0\\ 0, & \mathrm{otherwise} \end{array}\right. \end{array}$$

(21) $$\begin{array}{c} L_{\mathrm{MoE}}={\mathrm{CV}}^{2}\left(\sum_{b=1}^{B}G_{bi}\right)+{\mathrm{CV}}^{2}\left(\sum_{b=1}^{B}{\mathrm{GTL}}_{bi}\right) \end{array}$$

${CV}^{2}$ denotes the squared coefficient of variation. $G_{bi}$ denotes the gate weight of the *b*-th batch sample for the i-th expert.

The total loss is computed as follows.

(22) $$\begin{array}{c} L_{\mathrm{Total}}=L_{\mathrm{rec}}+\lambda L_{\mathrm{Per}}+\gamma L_{\mathrm{MoE}}, \end{array}$$

where λ=0.0001, and γ=5.

## 4. Experimental Results

### 4.1. Datasets and Setting

Dataset Configurations: This work uses two synthetic video deblurring datasets, DVD [36] and GoPro [44], as well as a real-world video deblurring dataset, BSD [17]. In BSD, different exposure times are set to obtain paired sharp and blurry videos, including 1ms–8ms, 2ms–16ms, and 3ms–24ms. Longer exposure times correspond to higher levels of blur. In the two synthetic datasets, blurry and sharp video pairs are synthesized from high-FPS sequences captured with devices such as the iPhone 6s, GoPro Hero 4 Black, and Canon 7D. In the BSD real-world dataset, Zhong et al. [17] employed a beam splitter system with two synchronized cameras, C1 and C2. The cameras are set to different exposure times to capture real blurry and sharp video pairs. To balance the irradiance between them, a neutral density filter is placed in front of C1. In our experiments, we use the 1ms–8ms and 3ms–24ms settings to compare the network’s performance on videos with different blur levels. The configurations of the three datasets are summarized in Table 2.

Configurations of Our Proposed Network: The CATF framework is implemented in PyTorch, using an NVIDIA GeForce RTX 4090 GPU on a Windows system. In our experiments, we set $N_{1}=5$, $N_{2}=5$, $N_{3}=25$, $M_{1}=1$, $M_{2}=1$, $M_{3}=1$, T=1, S=3, C=256, p=4, w=4, v=3 (one shallow expert, one medium expert, and one deep expert), k=2, $E_{1}=1000$, and $E_{2}=1500$.

Implementation Details: The training details are summarized in Table 3. We adopt the same training strategy as VDTR [21], including the optimizer settings and learning rate schedule. The MoNAF module and its corresponding loss $L_{\mathrm{MoE}}$ are excluded from training during the first 1000 epochs and are activated in the subsequent 1000 epochs.

### 4.2. Comparison with Video Deblurring Methods

STFAN (ICCV2019) [38], EDVR (CVPRW2019) [22], CDVDTSP (CVPR2020) [45], FGST (ICML2022) [46], STDANet (ECCV2022) [20], VDTR (TCSVT2023) [21], LightVID (TCSVT2024) [32], STCT (TIP2024) [24], and ALK-MoE (TCSVT2025) [47] are selected for the comparison experiments. SSIM [48] and PSNR [49] are utilized for quantitative comparison.

#### 4.2.1. Quantitative Comparison

As shown in Table 4, on the DVD test set, the proposed method achieves higher PSNR than most existing methods, with only a slight decrease compared to ALK-MoE. Although the SSIM scores are slightly lower than those of FGST, LightVID, and ALK-MoE, the differences are very small, and the scores are quite close. As shown in Table 5, on the GoPro test set, the proposed method achieves PSNR and SSIM scores that are slightly lower than but still comparable to the state-of-the-art ALK-MoE and higher than those of the other competing methods. As shown in Table 6, on the BSD test set, while the proposed method underperforms ALK-MoE by 0.56 dB in PSNR and 0.0019 in SSIM on the 1ms–8ms exposure test set, it exceeds ALK-MoE in SSIM by 0.0081 on the 3ms–24ms exposure test set, demonstrating its potential to handle complex scenarios. Table 7 presents the GFLOPs and running time of the five algorithms achieving the best PSNR and SSIM on the DVD and GoPro test sets. Although our method shows slightly lower PSNR and SSIM compared with ALK-MoE, it remains highly efficient, with GFLOPs 18% lower and the lowest running time among the compared methods.

#### 4.2.2. Qualitative Comparison

**Figure 4:**
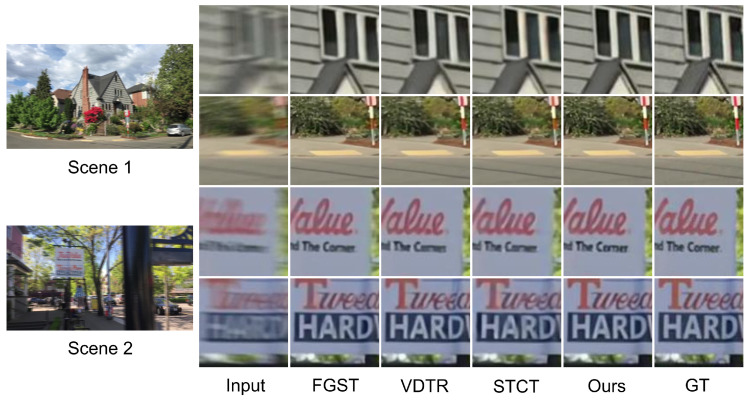
Visual results of DVD test set on different methods.

**Figure 5:**
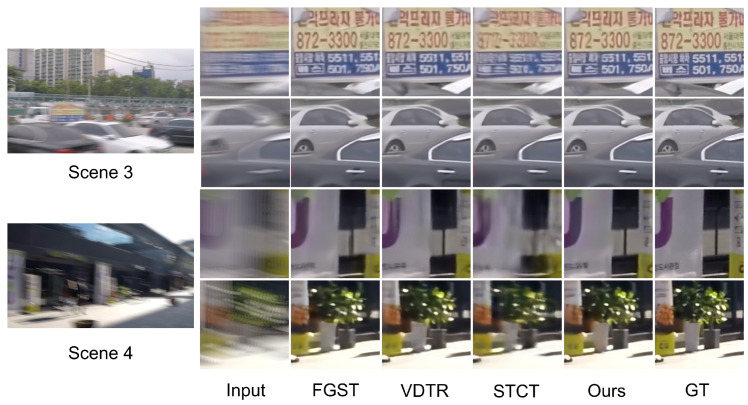
Visual results of GoPro test set on different methods.

**Figure 6:**
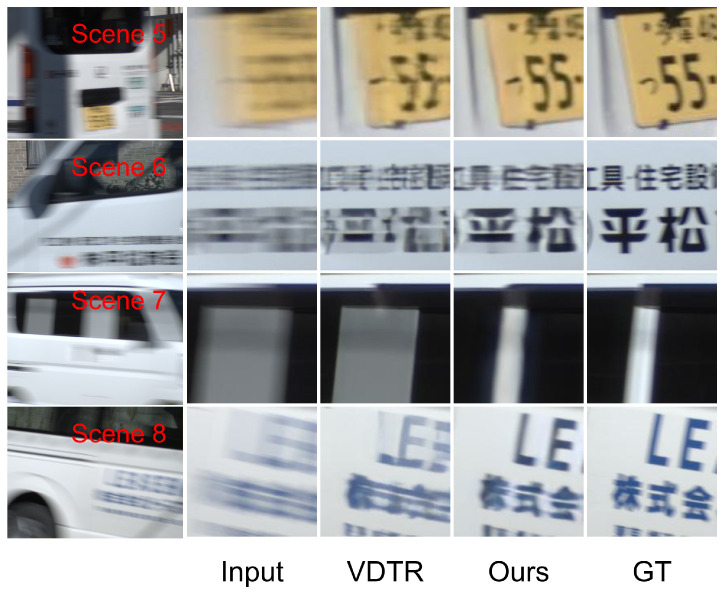
Visual results of BSD test set on different methods.

We select algorithms with similar quantitative performance for qualitative comparison: FGST, VDTR, STCT, and ALK-MoE on GoPro and DVD, and VDTR and ALK-MoE on BSD. ALK-MoE is excluded from qualitative evaluation, as it is not open-sourced. Scenes 1 and 2 (Figure 4) are drawn from the DVD test set, Scenes 3 and 4 (Figure 5) from the GoPro test set, Scenes 5 and 6 (Figure 6) from the 1 ms–8 ms exposure test set, and Scenes 7 and 8 (Figure 6) from the 3 ms–24 ms exposure test set. In Scenes 1 and 2, while all methods reconstruct structures well, our method achieves superior performance in detail and contrast. In Scenes 3 and 4, STCT fails to restore structures in extremely blurred regions, likely because its optical flow estimation module does not work well in such areas. VDTR can recover structures but performs poorly on fine details, such as the text in Scene 3. This is because VDTR is Transformer-based, and Transformers mainly aggregate features, while convolutional modules are better suited for high-frequency feature processing. FGST performs well in both structure and detail restoration due to its parallel architecture, which allows the network to utilize long-range feature information. However, FGST aligns and fuses neighboring features via flow guided attention, which causes error accumulation. Therefore, its detail restoration remains less effective than our method. In Scenes 5–8, due to the high relative velocity, VDTR fails to reconstruct fine details and structures, whereas our method succeeds.

### 4.3. Evaluation of Error Accumulation Mitigation and Parallelism

We design four groups of experiments to verify the ability of our proposed algorithm to suppress error accumulation in temporal dependency inference and its suitability for parallel computation.

Group 1 and Group 2: in the preparation phase, the input at the “previous-frame deblurred result” position is set to either the previous frame, or the GT of the previous frame, to verify whether the algorithm can suppress error accumulation in temporal dependency inference.

Group 3: the previous-frame deblurred result is replaced with the GT of the previous frame throughout all phases, to evaluate the degree of error accumulation in temporal dependency inference.

Group 4: the previous-frame is used throughout all phases, to assess the algorithm’s capability for temporal parallel computation.

As shown in Table 8, in Group 1, the PSNR is only 0.03 dB higher than in Group 2, indicating that the algorithm can adaptively adjust according to the quality of the previous frame’s reconstruction, demonstrating effective mitigation of error accumulation. In Group 3, there is no error accumulation, and compared with Groups 1 and 2, the PSNR increases by only 0.25 dB and 0.22 dB, respectively, indicating that the degree of error accumulation is very small. In Group 4, the PSNR is 0.95dB lower than Group 1. It still outperforms two methods in Table 5. In PSNR, it is only 0.01dB below STDANet and 0.12dB below LightVID, demonstrating the feasibility of parallel computation.

### 4.4. Ablation Experiment

As shown in Table 9, when the reconstruction of neighboring frames is removed and only the current frame is used for deblurring, the PSNR drops by more than 1 dB compared to CATF. This indicates that the features of both the current frame and neighboring frames are effectively utilized in CATF. Among the three neighboring frames $R_{t-1}$, $F_{t+1}^{input}$, and $F_{t+2}^{input}$, the reconstruction of $F_{t+1}^{input}$ achieves a PSNR that is 0.08 dB higher than that of $R_{t-1}$. This difference is not further investigated in this work, and the results in Table 4, Table 5 and Table 6 are based on the reconstruction of $F_{t+1}^{input}$.

As shown in Table 10, when using homogeneous experts, each with one Part 1 layer results in lower PSNR and SSIM but achieves faster inference than using two Part 1 layers. With heterogeneous experts adopting different structures, MoNAF achieves higher PSNR and SSIM than homogeneous experts with one Part 1 layer, and faster inference than those with two Part 1 layers. Although MoNAF introduces a modest overhead compared to the plain baseline without MoE, it strikes a better balance between deblurring quality and inference speed, demonstrating the benefit of input-adaptive expert selection.

### 4.5. Discussion

In this work, we only use PSNR and SSIM for quantitative comparison to remain consistent with prior studies and enable direct comparisons. However, PSNR and SSIM do not always correlate well with human perceptual quality and may not fully reflect improvements in visual appearance. To more comprehensively evaluate the perceptual quality of the restored results, future work will consider incorporating deep learning–based perceptual metrics, such as LPIPS [50] and CLIP-IQA [51].

As shown in Figure 6, CATF performs better than the compared methods in extremely fast motion scenes, but the deblurred results mainly recover coarse structures, and the fine detail reconstruction remains poor. In future work, we will explore event camera based deblurring, and fuse high temporal resolution motion cues with frame images, to improve detail recovery in fast motion scenarios.

Under the 1280×720 setting on an NVIDIA RTX 4090, CATF takes 198.8 ms per frame, which corresponds to 5.03 FPS. Under this setting, CATF does not meet real-time requirements. On the BSD dataset, where the input resolution is 640×480, CATF achieves 54.6 ms per frame, which corresponds to 18.31 FPS. Since smooth real-time performance typically requires about 30 FPS, these results indicate that CATF can approach real-time speed when the input resolution is sufficiently small. However, CATF still falls far short of the 240–1000 FPS demanded by high-speed camera applications; so, the current implementation does not yet satisfy such strict real-time constraints. Nevertheless, compared with existing video deblurring methods, CATF achieves a more favorable trade-off between computational cost and deblurring performance.

## 5. Conclusions

Image sensing tasks are often applied in high-speed scenarios, where the captured images tend to suffer from blur, thereby degrading the sensing accuracy. Based on this, this paper proposes a video deblurring algorithm tailored for image sensing tasks. To achieve high-quality deblurring, fast inference, and wide applicability, this paper proposes a Current-Aware Temporal Fusion with Input-Adaptive Heterogeneous Mixture-of-Experts method. First, single-stream networks are prone to interference from irrelevant neighboring-frame features. The shared extractor cannot fully exploit the distinct cues of the current and neighboring frames. To address this issue, we propose the CATF structure, which employs a dual-stream design and processes the current frame and neighboring frames separately according to the characteristics of convolution and MSA, thereby avoiding interference and fully leveraging the current frame information. Furthermore, considering the large variation in blur across video frames, we design the MoNAF module, which contains heterogeneous experts of varying depths and can select appropriate experts based on the blur level of each frame, thus reducing the inference time while maintaining the deblurring quality. Finally, we propose a training strategy that can both suppress error accumulation under temporally dependent inference and achieve satisfactory results in parallel computation scenarios, thereby enhancing the method’s wide applicability.

We conduct extensive quantitative and qualitative experiments on the DVD, GoPro, and BSD datasets. The quantitative results (Table 4 and Table 5) show that CATF achieves PSNR and SSIM close to those of the latest method ALK-MoE on DVD and GoPro, although slightly lower. On BSD (Table 6), although CATF yields a 0.56 dB lower PSNR than ALK-MoE under the 1 ms–8 ms exposure setting, it achieves higher SSIM under the more challenging 3 ms–24 ms exposure setting, indicating stronger structural fidelity under severe blur. In addition, CATF achieves the shortest average inference time per frame (Table 7), demonstrating a favorable balance between deblurring quality and computational efficiency. Qualitative comparisons (Figure 4, Figure 5 and Figure 6) further confirm that CATF restores image structures and details effectively. Moreover, we validate the feasibility of CATF under both sequential and parallel computation settings (Table 8), and further verify the effectiveness of the CATF architecture and the MoNAF module (Table 9 and Table 10). The results of this paper provide an important supporting technology for image sensing.

## Figures and Tables

**Table 1:** Comparison of video deblurring methods based on different input types.

Method	Key Advantages	Main Limitations
Sliding Window-Based	Fuses temporal information across frames	Limits feature reuse across frames; restricts long-range modeling due to window size
Recurrent	Shares parameters across frames; supports feature reuse; fuses temporal information across frames	Cannot perform parallel computation; accumulates errors over time; limits long-range modeling; performs poorly on few-frame videos [33]
Parallel	Supports multi-frame parallel processing; fuses temporal information across frames; supports feature reuse	Requires a large model; consumes high memory; constrains scalability by hardware resources

**Table 2:** The configurations of the three datasets.

Dataset	DVD		GoPro		BSD		
	Train	Test	Train	Test	Train	Val	Test
Videos	61	10	22	11	60	20	20
Frames	5708	1000	2103	1111	6000	2000	3000

**Table 3:** Training settings.

Component	Setting
Data augmentation	Random crop (256 × 256), horizontal flips, vertical flips
Optimizer	ADAM
ADAM parameters	$\beta_{1}=0.9$, $\beta_{2}=0.99$
Initial learning rate	$4\times 10^{-4}$ (DVD and GoPro) $2\times 10^{-4}$ (BSD)
Epoch	2000
Batch size	5

**Table 4:** Evaluations on the DVD test set in terms of PSNR and SSIM. Values from FGST and LightVID are reported to three decimal places. The highest scores are highlighted in bold, while the second-highest scores are indicated with an underline.

Metric	STFAN	CDVDTSP	FGST	STDANet	VDTR
PSNR	31.15	32.13	33.50	33.05	33.13
SSIM	0.9049	0.9268	0.945	0.9374	0.9359
**Metric**	**LightVID**	**STCT**	**ALK-MoE**	**Ours**	
PSNR	32.51	33.45	**33.63**	33.54	
SSIM	**0.946**	0.9421	0.9448	0.9430	

**Table 5:** Evaluations on the GoPro test set in terms of PSNR and SSIM. Values from FGST and LightVID are reported to three decimal places. The highest scores are highlighted in bold, while the second-highest scores are indicated with an underline.

Metric	STFAN	CDVDTSP	FGST	STDANet	VDTR
PSNR	28.59	31.67	33.02	32.62	33.15
SSIM	0.8608	0.9279	0.947	0.9375	0.9402
**Metric**	**LightVID**	**STCT**	**ALK-MoE**	**Ours**	
PSNR	32.73	32.97	**33.79**	33.56	
SSIM	0.941	0.9406	**0.9516**	0.9477	

**Table 6:** Evaluations on the BSD test set in terms of PSNR and SSIM. The highest scores are highlighted in bold, while the second-highest scores are indicated with an underline.

Method	1 ms–8 ms		3 ms–24 ms	
	PSNR	SSIM	PSNR	SSIM
STFAN	32.78	0.9219	29.47	0.8716
EDVR	33.16	0.9325	31.93	0.9261
CDVDTSP	33.54	0.9415	31.58	0.9258
VDTR	34.12	0.9436	32.53	0.9363
ALK-MoE	**35.12**	**0.9505**	**33.42**	0.9372
Ours	34.56	0.9486	33.09	**0.9453**

**Table 7:** Comparison of model GFLOPs and average per-frame inference time on the GoPro test set (input resolution: 1280×720). The lowest GFLOPs and the shortest average per-frame inference time are highlighted in bold. The inference time of ALK-MoE is not reported because its code is not publicly available.

Method	GFLOPs (G)	Time (ms)
FGST	2075.1	1011.9
VDTR	2244.7	266.7
STCT	44,620.5	1239.0
ALK-MoE	1650.0	–
Ours	**1352.1**	**198.8**

**Table 8:** Evaluation of error accumulation mitigation and parallelism on the GoPro test set.

Group	Previous-Frame Deblurred Result (Preparation Phase, 4 Frames) Replaced with	Previous-Frame Deblurred Result (Actual Phase) Replaced with	PSNR	SSIM
Group 1	GT	Deblurred result	33.56	0.9477
Group 2	Previous frame	Deblurred result	33.53	0.9475
Group 3	GT	GT	33.78	0.9492
Group 4	Previous frame	Previous frame	32.61	0.9378

**Table 9:** The ablation experiments of the CATF architecture on the GoPro test set in terms of SSIM and PSNR.

Method	PSNR	SSIM
CATF (Current-frame reconstruction and w/o MoNAF)	31.82	0.9262
CATF (w/o MoNAF) (Reconstruct $R_{t-1}$)	33.11	0.9432
CATF (w/o MoNAF) (Reconstruct $F_{t+1}^{\mathrm{input}}$)	33.19	0.9438

**Table 10:** The MoNAF ablation experiments on the GoPro test set in terms of SSIM and PSNR. “2 Part 1” refers to two layers of Part 1 modules.

Type	The Numbers of MoE	Expert 1	Expert 2	Expert 3	PSNR	SSIM	FPS
None	None	None	None	None	33.11	0.9432	5.40
Homogeneous	3	Part 1	Part 1	Part 1	33.29	0.9456	4.77
Homogeneous	3	2 Part 1	2 Part 1	2 Part 1	33.38	0.9466	4.56
Heterogeneous	3	Part 1	Part 2	NAFBlock	33.31	0.9456	4.88

## Data Availability

Anyone can use or modify the source code for academic purposes only. The code is publicly available at: https://github.com/ZHANGYW1/CATF, accessed on 30 December 2025.
